# Supplementary figures and images for: Isolating and quantifying the role of developmental noise in generating phenotypic variation
Source: PLoS Comput Biol. 2019 Apr 22;15(4):e1006943. doi: 10.1371/journal.pcbi.1006943 (PMC6497311; doi:10.1371/journal.pcbi.1006943)

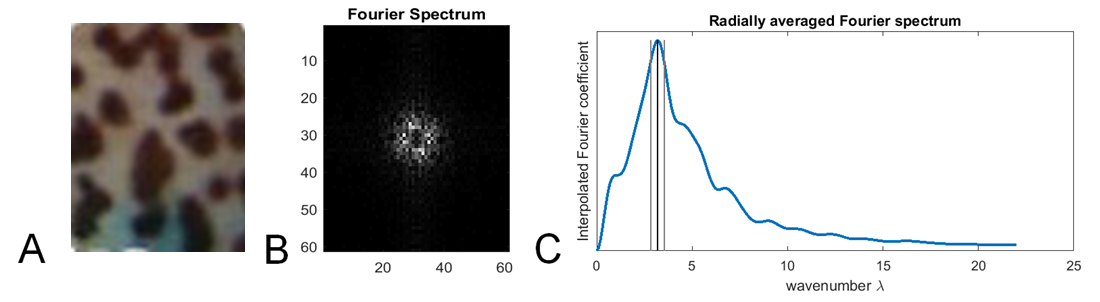

Supplement: S1 Fig — A: a rectangular section of the pattern for Gecko ID #731. B: the corresponding centered Fourier spectrum. Low-frequency components are shown in the center of the image, high-frequency components on the edges. Lighter colors indicate larger values. C: a plot of radially averaged interpolated absolute Fourier coefficient as a function of radius λ. The vertical lines indicate the location of the wavenumber corresponding to the maximum interpolated Fourier coefficient, as well as the interval in which its value is within 90% of the maximum. (TIFF) [file pcbi.1006943.s001.tiff]

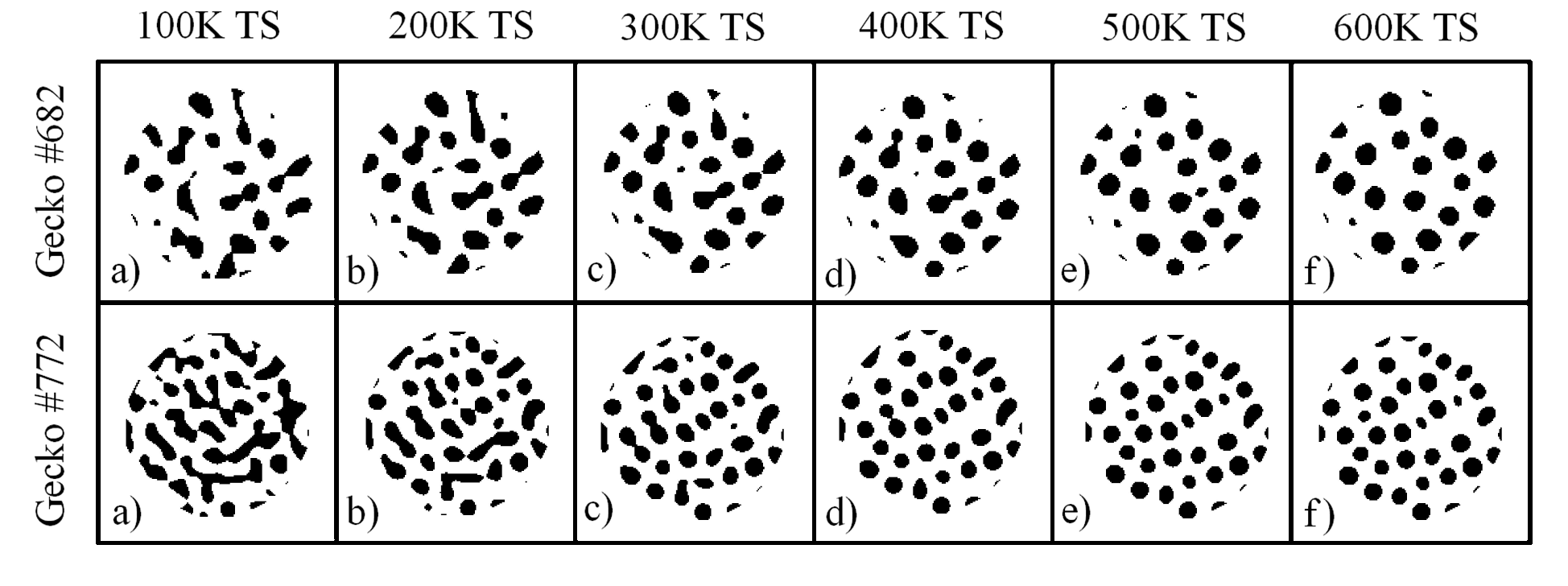

Supplement: S2 Fig — The panels (a-f) show the pattern every 100K time steps for a simulation with parameters corresponding to those of Gecko #682. The LALI-type for Gecko #682 is matched at 200K time steps. (TIFF) [file pcbi.1006943.s002.tiff]

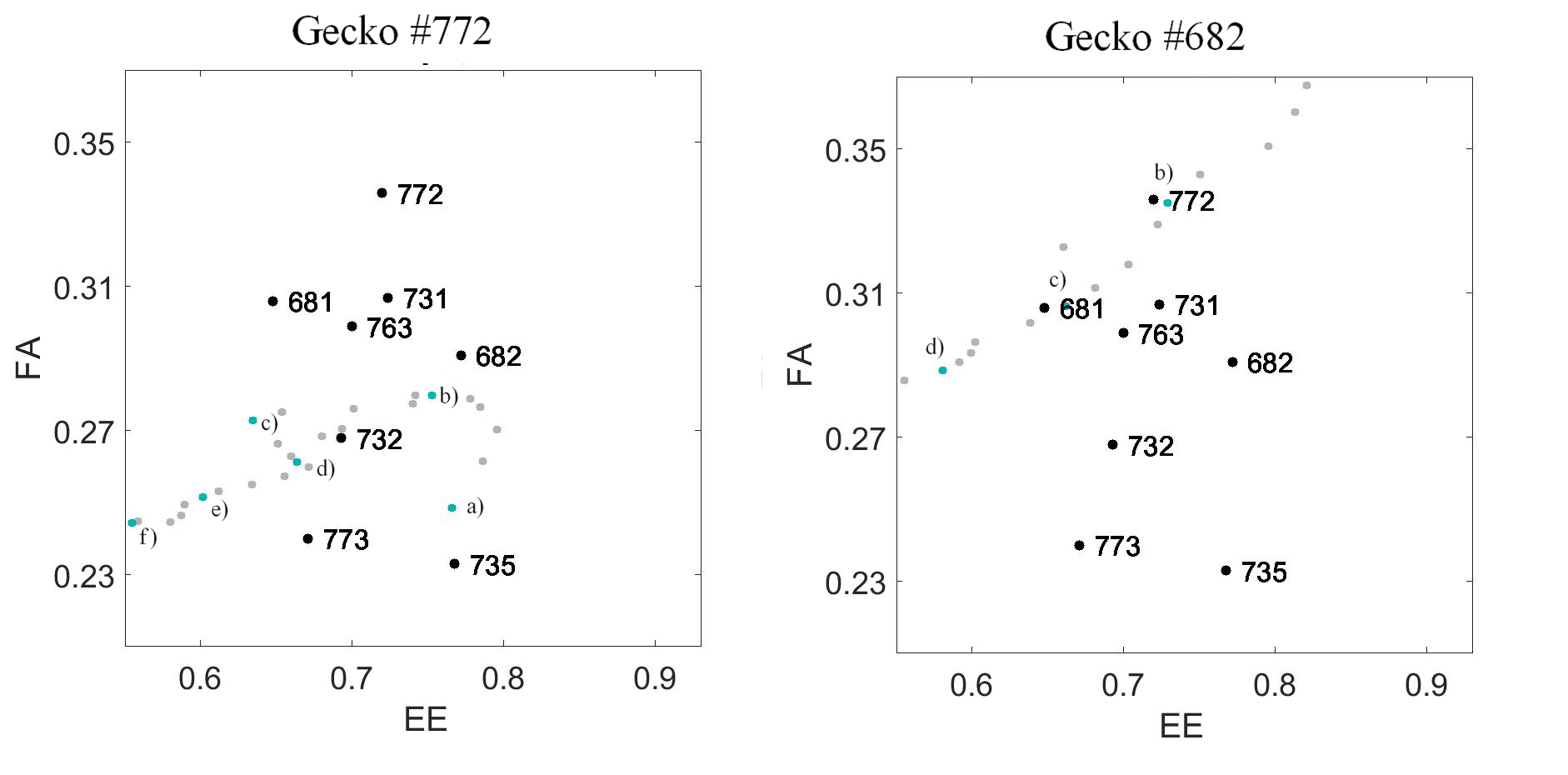

Supplement: S3 Fig — As the simulation progresses, the morphological properties of the spotted pattern change and create a path through FA-EE phenotype space. The points labeled (a-f), when they appear on the frame, correspond to the panels (a-f) described in S2 Fig. (TIFF) [file pcbi.1006943.s003.tiff]

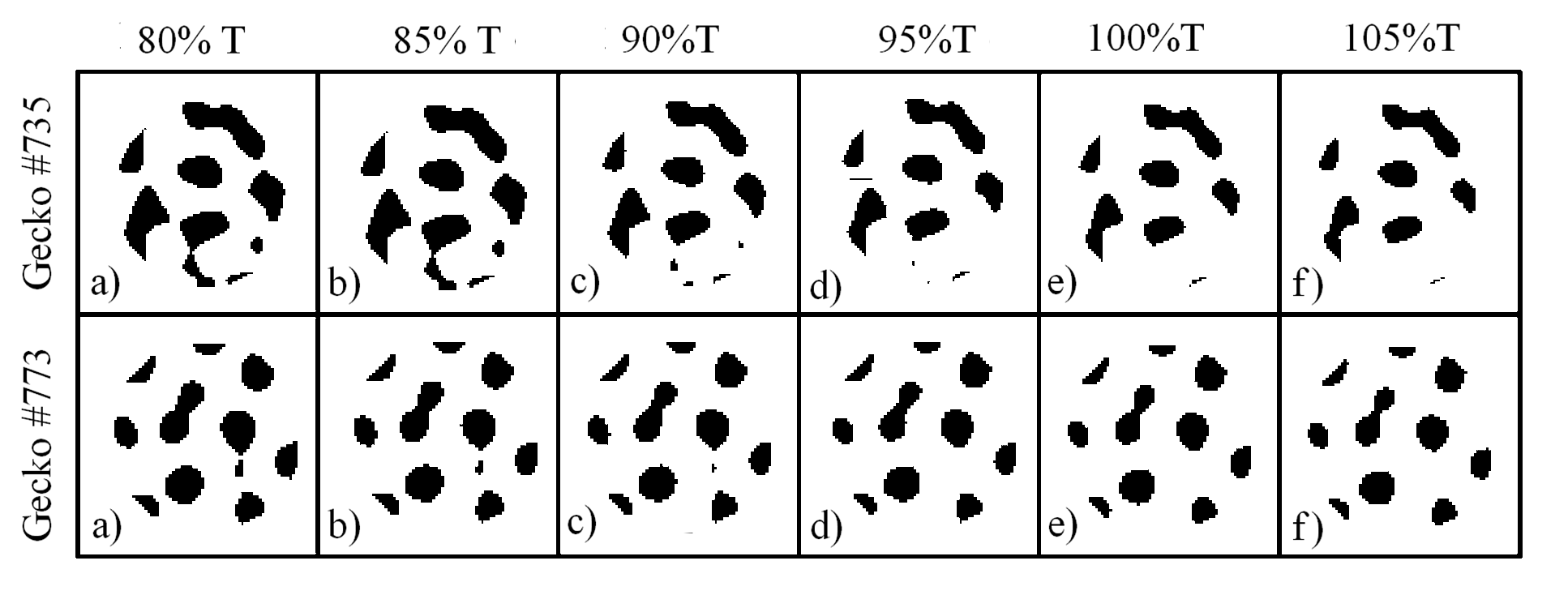

Supplement: S4 Fig — The panels (a-f) show the pattern every as the threshold varied from 80% to 105% in 5% increments for a simulation with parameters corresponding to those of Gecko #682. The LALI-type for Gecko #682 is matched when the threshold is 100%. (TIFF) [file pcbi.1006943.s004.tiff]

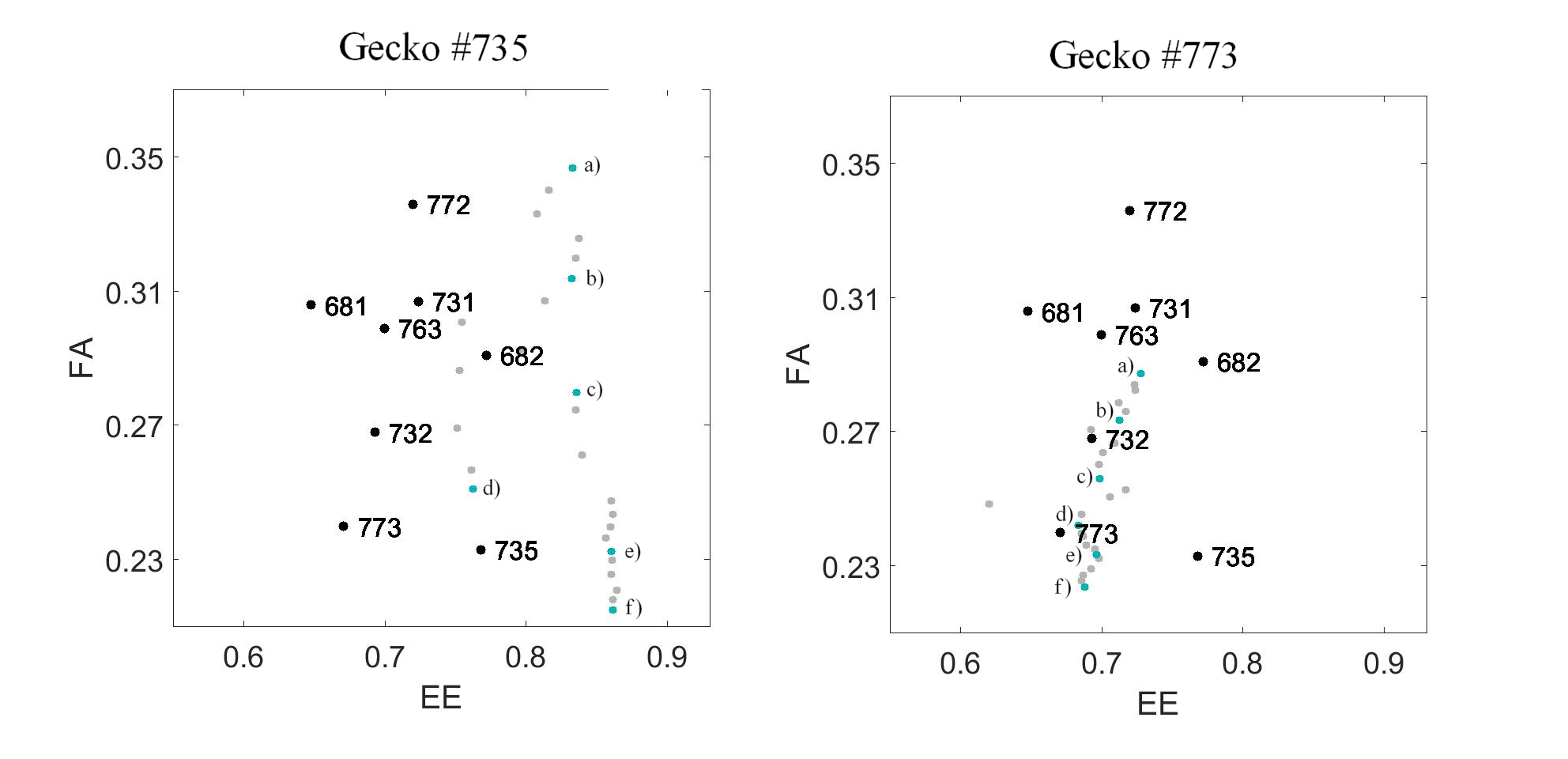

Supplement: S5 Fig — As the simulation progresses, the morphological properties of the spotted pattern changes and create a path through FA-EE phenotype space. The points labeled (a-f), when they appear on the frame, correspond to the panels (a-f) described in S4 Fig. (TIFF) [file pcbi.1006943.s005.tiff]
